# Feasibility of medial parapatellar approach in unicompartmental knee arthroplasty for moderate to severe varus deformity

**DOI:** 10.1007/s00402-025-06183-2

**Published:** 2026-02-02

**Authors:** Shihua Zou, Lijun Xiang, Hong Liu, Ming Ji, Xiaojiang Xiong, Tao Yang

**Affiliations:** 1 Department of Orthopedics, Chongqing University Affiliated Three Gorges Hospital, 165 Xincheng Road, Wanzhou District, Chongqing City, 404199 People’s Republic of China; 2 Department of Central Operating Room, Chongqing University Affiliated Three Gorges Hospital, 165 Xincheng Road, Wanzhou District, Chongqing City, 404199 People’s Republic of China

**Keywords:** Unicompartmental knee arthroplasty (UKA), Moderate to severe varus knee osteoarthritis, Medial parapatellar approach

## Abstract

**Research background and purpose:**

Unicompartmental knee arthroplasty (UKA) is well-established for mild varus deformity, but its application in moderate to severe varus cases remains technically challenging. This study aims to preliminarily evaluate whether adopting the traditional medial parapatellar approach in UKA procedures for these complex deformities can yield acceptable early outcomes.

**Methods:**

A retrospective study was conducted. Between January 2023 and March 2024, 9 patients with moderate to severe varus deformity underwent medial UKA using a cemented fixed-bearing Link prosthesis by medial parapatellar approach. The cohort included 1 male and 8 females with a mean age of 65.3 years and mean weight of 60.6 kg. All procedures were performed by a single surgeon. Preoperative and final follow-up assessments included bilateral full-length standing radiographs for hip-knee-ankle angle (HKA), The Angle between the femoral mechanical axis and the tibial mechanical axis (hip-knee-ankle angle, HKA) was measured by software and recorded. The hip-knee-ankle (HKA) angle was reported directly as the angle between the femoral and tibial mechanical axes. The normal alignment range was defined as 178°–182°, with values below 178° indicating varus deformity and those above 182° indicating valgus deformity. Knee range of motion (ROM), Hospital for Special Surgery (HSS) score, and Knee Society Score (KSS). Statistical analysis was performed using SPSS 26.0, with P < 0.05 considered statistically significant.

**Results:**

The follow-up of 15.20 ± 1.95 months, all patients demonstrated primary wound healing without perioperative complications. Significant improvements were observed in all measured parameters: HSS score improved from 50.11 ± 3.41 to 92.11 ± 2.37 95% CI: (40.12, 43.88); KSS score from 59.89 ± 3.55 to 88.78 ± 2.49 95% CI: (27.65, 30.13); KSS function score from 44.44 ± 6.82 to 76.67 ± 7.07 95% CI: (28.33,36.12); ROM from 94.22 ± 1.92° to 122.67 ± 2.83° 95% CI: (26.60, 30.29); and HKA from 164.58 ± 4.16° to 176.64 ± 2.20°95% CI: (10.16, 13.98). All improvements were statistically significant (P < 0.001). No cases of aseptic loosening, unexplained pain, or polyethylene liner dislocation were observed during follow-up.

**Conclusion:**

This preliminary experience suggests medial parapatellar approach for UKA in moderate to severe varus deformity may represent a technical option for carefully selected cases. However, these observations are limited by the small sample size and relatively short follow-up. Further validation through larger-scale studies with extended follow-up is warranted to establish long-term efficacy and safety.

## Research background

With evolving techniques in unicompartmental knee arthroplasty (UKA),which current options include minimally invasive, robotic-assisted, and conventional medial parapatellar approaches [1–4], While minimally invasive and robotic-assisted techniques have dominated recent UKA development [5]. The UKA is well-established for mild varus deformity [6], The application of unicompartmental knee arthroplasty (UKA) in patients with moderate to severe varus deformity (≥ 15°) remains a subject of considerable debate [7].

Severe varus deformity presents particular challenges for UKA, including difficulty in achieving neutral limb alignment and concerns regarding residual varus malalignment leading to medial compartment overload, accelerated polyethylene wear, and potential early failure [8–12]. But Seng [13] published the research shows that varus deformity ranged from 18° to 20°can achieve good function and quality of life (QOL) with UKA if satisfactory mechanical alignment is restored. Historically, these concerns have positioned severe varus as a relative contraindication to UKA, which may explain the limited investigation into the use of the more extensive medial parapatellar approach for such cases.

Medial parapatellar approach offers enhanced exposure of the posteromedial compartment, better visualization, balancing in tight posteromedial corners [12, 14].which potentially facilitating more accurate bone resection and soft tissue balancing in severe deformities [15]. However, this technique has not been systematically studied in the context of UKA for severe varus, primarily because such deformities have historically been considered relative contraindications to UKA, and surgical innovation has focused predominantly on minimally invasive and robotic techniques for conventional indications [16].

This study hypothesized that patients with severe varus deformity can achieve satisfactory early-stage outcomes following unicompartmental knee arthroplasty (UKA), provided the procedure is performed by a conventional medial parapatellar approach.

## Materials and methods

### General information

This study had a retrospective design and was approved by our hospital ethics committee (No.IIT2025-00103). Patients who underwent Link fixed platform unicompartmental knee arthroplasty in hospital from January 2023 to March 2024 were selected nine cases. Among them, there was 1 male with 1 knee and 8 females with 8 knees. The cohort had a mean age of 65.3 years (range 60–74 years) and a mean weight of 60.6 kg (range 48–74 kg). The medial UKA procedures were performed using a fixed-bearing Link prosthesis with cemented fixation. All operations were conducted by a single senior arthroplasty surgeon with over 10 years of experience. No formal sample size calculation was performed; the sample size was determined based on feasibility to meet the primary objectives of this pilot study.

**Fig. 1:**
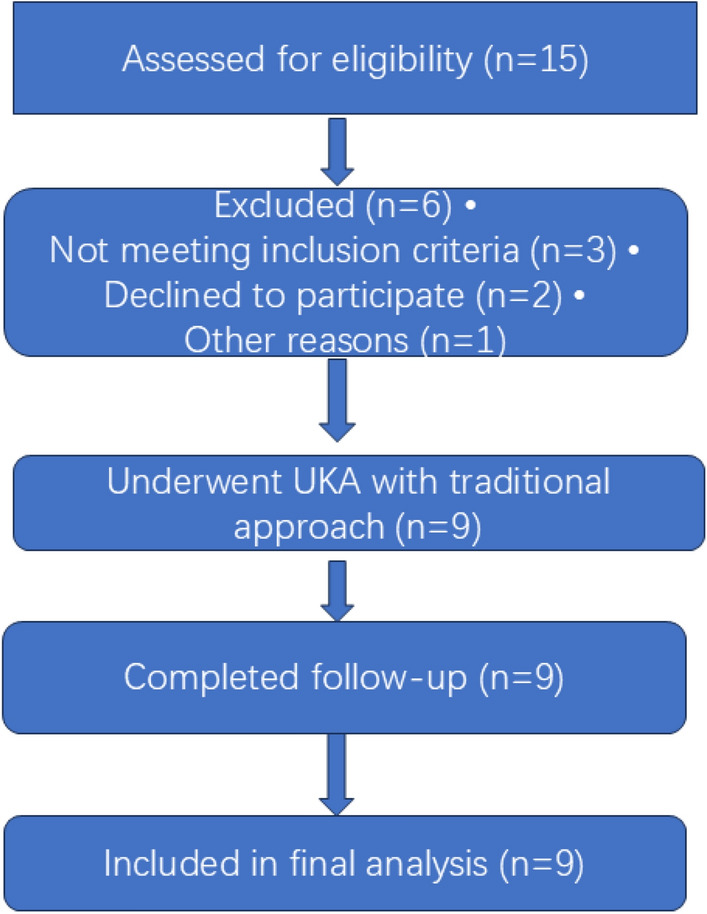
Patient Flow Diagram. Notes: This diagram follows CONSORT guidelines for clinical studies. All enrolled patients received the intended intervention. No patients were lost to follow-up during the study period. Final analysis was performed on per-protocol basis

Relevant information of the sample before operation (see Tables 1, 2 and Fig. 1):

**Table 1:** Study inclusion and exclusion criteria

Category	Criteria
Inclusion Criteria	(1) Symptomatic anteromedial osteoarthritis (2) Intact cruciate/collateral ligaments with medial joint line tenderness only (3) Range of motion > 90° with flexion contracture < 5° (4) Radiographic confirmation of isolated medial compartment disease (5) Absence of major organ dysfunction
Exclusion Criteria	(1) Flexion contracture > 10° (2) Participation in sports or heavy labor (3) Multi-compartment involvement or significant patellofemoral symptoms (4) Severe osteoporosis (5) Inflammatory or infectious arthritis (6) Radiographic evidence of lateral/patellofemoral degeneration or ACL deficiency (7).Opted for TKA

**Table 2:** Patient demographics

	Gender	Age (years)	Height (cm)	Weight (Kg)
Sample1	F	63	155	61
Sample2	M	64	168	67
Sample3	F	60	156	57
Sample4	F	61	155	56
Sample5	F	62	158	60
Sample6	F	70	160	61
Sample7	F	65	148	74
Sample8	F	69	158	48
Sample9	F	74	150	53

### Methods of treatment

The traditional TKA approach provides a critical advantage over minimally invasive and robotic-assisted techniques through its extensive parapatellar exposure. This enhanced visualization enables direct access to the posteromedial compartment, which proves essential for achieving precise ligament balancing, accurate component positioning, and effective deformity correction in severe varus knees.

It follow-up protocol was both clinical and radiological, as is standard for post-arthroplasty evaluation; Clinical assessments were performed by the attending orthopedic surgeon.These assessments included evaluation of knee range of motion, Hospital for Special Surgery (HSS) score, e.g., Implant alignment and the presence of radiolucent lines, were measured by software, two independent orthopedic surgeons to ensure reliability. To quantify reliability, both intra-observer and inter-observer consistencies were evaluated using intraclass correlation coefficients (ICCs), with values greater than 0.80 indicating excellent agreement. Any discrepancies in measurements were resolved through consensus or by adjudication of a third senior surgeon.

### Indicators of observation

(1) HSS score was used to evaluate knee function preoperation and last follow-up. HSS score: pain, function, range of motion, muscle strength, knee joint deformity, stability were scored, using the cane points, the lower the score, the worse the knee joint function. (2) knee society score (KSS) was used to evaluate knee function preoperation and last follow-up; The Knee Score and Function Score C were used to measure the maximum flexion Angle before and after operation. (3) the postoperative and anteroposterior of bilateral full-length standing lower extremity radiograph(AP view). Radiological assessments were conducted by a single blinded reviewer 5 patients > 15° varus. two investigators performed follow-up assessments. (4) There was no deep vein thrombosis, prosthesis loosening or dislocation, periprosthetic fracture, infection and other serious complications after operation.

### Statistical treatment

The data were imported into SPSS26.0 statistical software for data analysis. The measurement data were represented by ($\overline{x }$ ± s). Paired sample t test was used to compare the data before and after operation. P < 0.05 was considered statistically significant, P > 0.05 was not statistically significant, and P < 0.01 was statistically significant. A potential limitation of the present study lies in the execution of multiple t-tests without appropriate statistical correction, which may elevate the risk of Type I error. The distribution of HKA improvement was illustrated using a scatter plot by GraphpadPrism 9.0

Comparison of knee joint function and imaging evaluation results before operation and at the last follow-up ($\overline{x }$ ± s) (see Table 3).

**Table 3:** Preoperative and Postoperative Outcomes

Time			Hss		KSS	KSS (Function)		ROM	HKA	
Pre-operation			50.11 ± 3.41		59.89 ± 3.55	44.44 ± 6.82		94.22 ± 1.92	164.58 ± 4.16	
Preoperative Range(min–max)			(43,55)		(53,65)	(30,50)		(90,96)	(154.6,168.5)	
Post-operation			92.11 ± 2.37		88.78 ± 2.49	76.67 ± 7.07		122.67 ± 2.83	176.64 ± 2.20	
Postoperative Range(min–max)			(88,95)		(83,92)	(60,80)		(120,127)	(172.8,179.2)	
t			51.44		53.63	19.07		35.50	14.58	
P			0.001		0.001	0.001		0.001	0.001	
95%CI			(40.12,43.88)		(27.65,30.13)	(28.33,36.12)		(26.60,30.29)	(10.16,13.98)	
Cohen's d			17.15		17.88	6.36		11.83	4.86	

**Fig. 2:**
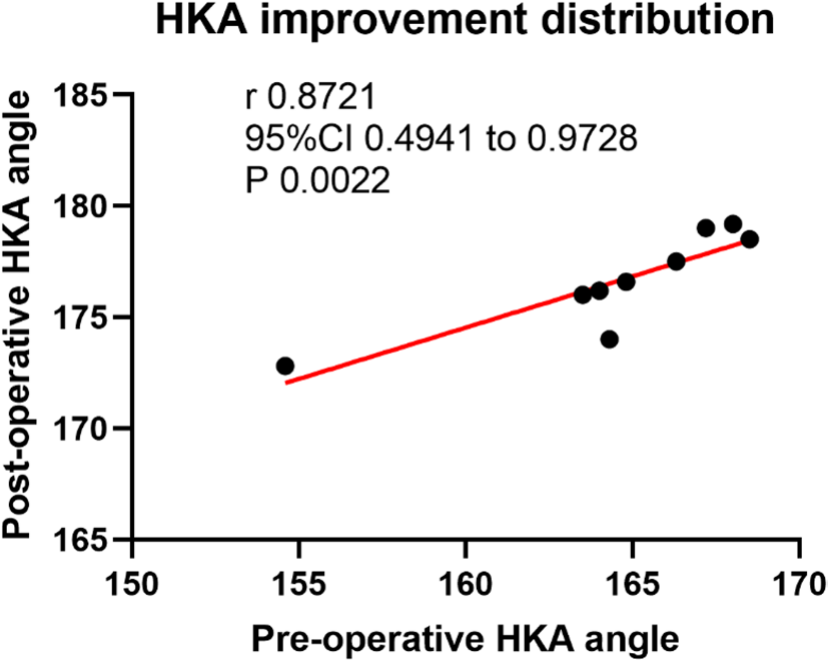
The scatter plot of HKA improvement distribution. The preoperative HKA was (164.58 ± 4°) and postoperatively (177.64 ± 2.20°); The results of scatter plot showed that the r value was 0.87; 95%CI (0.49, 0.97), P = 0.001,  < 0.05

The maximal knee joint activity increased from preoperative (94.22 ± 1.92°) to postoperative (122.67 ± 2.83°). The preoperative and postoperative data compared the differences were statistically significant (P < 0.05). Tzhe surgical incisions of the patients achieved Grade A healing, and no perioperative complications such as infection, pulmonary embolism, deep venous thrombus of the lower extremities, iatrogenic neurovascular injury, or periprosthetic fractures occurred. The follow-up period was 15.20 ± 1.95 months (ranging 12.00 from 18.00 months). The quality of life of the patients was significantly improved compared with that before the operation. None of the cases had complications such as aseptic prosthesis loosening, pain of unknown cause, or dislocation of polyethylene pads (see Fig. 2).

## Discussion

The scatter plot of HKA improvement distribution indicates a very strong, direct relationship between the pre-operative and post-operative HKA angles. As the pre-operative angle increases or decreases, the post-operative angle changes in the same direction in a very predictable pattern. The surgeon's technique was highly consistent. Regardless of the severity of the initial deformity (whether the knee was very bow-legged or knock-kneed), the surgical correction followed a reliable formula. This reduces outcome variability and increases the surgeon's confidence in achieving the planned mechanical alignment. The probability that this strong correlation occurred purely by chance is very low (only 0.22%). Therefore, the observed relationship is considered statistically significant and reflects a true association in the patient population. The finding is reliable and not a fluke**.** Surgeons and researchers can be confident that the relationship is real and can be applied when planning future surgeries. The scatter plot doesn't just show a correlation; it shows that the surgical correction was systematic. The patients who started with a more severe deformity (e.g., an HKA of 163.5°) were corrected to a similar final angle as those who started with a milder deformity (e.g., an HKA of 168.5°). This demonstrates that the surgery effectively reduces the variability in knee alignment, grouping most patients around the desirable post-operative target.

This case series demonstrates the technical feasibility of performing fixed-bearing unicompartmental knee arthroplasty (UKA) by medial parapatellar approach in a highly selected cohort of patients with moderate to severe varus deformity. The procedure resulted in significant coronal plane correction, with a strong, predictable correlation between preoperative and postoperative alignment (p < 0.01), indicating a systematic and consistent surgical technique. Short-term follow-up showed satisfactory outcomes and soft-tissue balance in all cases, despite the presence of residual varus alignment in some patients.

Our findings contribute to the ongoing debate regarding the indications and limits of UKA in varus deformity. The studies reporting satisfactory short-term to medium-term outcomes with fixed-bearing UKA, even in knees with substantial medial compartment osteoarthritis or stable anterior cruciate ligament deficiency [17, 18].The observation that moderate residual varus (e.g., 6.6° in our most severe case) can be compatible with good short-term results is supported by some literature [19, 20]. Furthermore, recent evidence suggests that joint line obliquity may not adversely affect functional outcomes at medium-term follow-up [21, 22].

However, our findings also intersect with significant concerns in the literature. Severe preoperative varus is traditionally considered a relative contraindication for UKA due to challenges in restoring neutral alignment, risks of medial compartment overload, polyethylene wear, and potential progression of lateral compartment disease [23–26]. Biomechanical studies consistently indicate that varus alignment increases medial compartment loading [24, 25, 27], and clinical series identify varus alignment as a risk factor for revision [12, 27]. The medial edge loading observed in some of our cases, while not leading to early failure, mirrors these biomechanical concerns and underscores the potential threat to long-term implant survivorship. Clinical Relevance: Feasibility The primary clinical relevance of this study is confined to establishing proof-of-concept feasibility. It indicates that in exceptional cases of severe, compensated varus malalignment, UKA can be performed through a standard TKA exposure without immediate catastrophic failure. This approach allowed for adequate visualization, osteophyte removal, and controlled medial soft-tissue release. The case with 25.4° of preoperative varus correction highlights the potential upper limit of deformity that might be addressed, but it simultaneously serves as a cautionary example due to the resultant residual varus and edge loading. Therefore, this technical strategy should be viewed as an option for highly specific, complex scenarios rather than a rationale for expanding standard UKA indications to moderate or severe varus.

This study has significant limitations that necessitate cautious interpretation of the findings: The series includes only 9 knees, which severely limits the statistical power, robustness of any conclusions, and generalizability of the results. The retrospective, single-surgeon, single-center case series design without a control group introduces inherent biases and prevents causal inferences. The reported outcomes are short-term, capturing feasibility and early safety but providing no information on long-term survivorship, polyethylene wear, or progression of arthritis in other compartments. Given the minuscule cohort, the validity of reported p-values is questionable, and findings should be considered preliminary observations rather than definitive statistical evidence.

The preliminary nature of these findings highlights the need for more rigorous investigation. Future studies should prioritize: Larger, multicenter prospective trials to adequately evaluate safety, efficacy, and optimal patient selection criteria; Longitudinal studies with extended follow-up to assess long-term implant survivorship, functional outcomes, and complication rates in this challenging population; Controlled comparisons, such as randomized controlled trials or propensity-matched analyses, directly comparing UKA with TKA in severe varus deformity to establish the most effective surgical strategy.

Conclusion Although the preliminary findings of this study provide valuable reference, they underscore the need for larger-scale, multi-center, prospective cohort studies or randomized controlled trials. Such investigations are essential to further validate the feasibility, long-term safety, and functional outcomes of applying this surgical strategy in patients with moderate-to-severe varus deformity.

## Data Availability

No datasets were generated or analysed during the current study.
